# Connectivity Measures Differentiate Cortical and Subcortical Sub-Acute Ischemic Stroke Patients

**DOI:** 10.3389/fnhum.2021.669915

**Published:** 2021-07-01

**Authors:** Chiara Fanciullacci, Alessandro Panarese, Vincenzo Spina, Michael Lassi, Alberto Mazzoni, Fiorenzo Artoni, Silvestro Micera, Carmelo Chisari

**Affiliations:** ^1^The BioRobotics Institute, Scuola Superiore Sant’Anna, Pisa, Italy; ^2^Unit of Neurorehabilitation, Department of Medical Specialties, University Hospital of Pisa, Pisa, Italy; ^3^Translational Neural Engineering Laboratory, Center for Neuroprosthetics, Institute of Bioengineering, École Polytechnique Fédérale de Lausanne (EPFL), Lausanne, Switzerland

**Keywords:** stroke, qEEG, brain power, connectivity, network theory

## Abstract

Brain lesions caused by cerebral ischemia lead to network disturbances in both hemispheres, causing a subsequent reorganization of functional connectivity both locally and remotely with respect to the injury. Quantitative electroencephalography (qEEG) methods have long been used for exploring brain electrical activity and functional connectivity modifications after stroke. However, results obtained so far are not univocal. Here, we used basic and advanced EEG methods to characterize how brain activity and functional connectivity change after stroke. Thirty-three unilateral post stroke patients in the sub-acute phase and ten neurologically intact age-matched right-handed subjects were enrolled. Patients were subdivided into two groups based on lesion location: cortico-subcortical (**CS**, *n* = 18) and subcortical (**S**, *n* = 15), respectively. Stroke patients were evaluated in the period ranging from 45 days since the acute event (T0) up to 3 months after stroke (T1) with both neurophysiological (resting state EEG) and clinical assessment (Barthel Index, BI) measures, while healthy subjects were evaluated once. Brain power at T0 was similar between the two groups of patients in all frequency bands considered (δ, θ, α, and β). However, evolution of θ-band power over time was different, with a normalization only in the **CS** group. Instead, average connectivity and specific network measures (*Integration*, *Segregation*, and *Small-worldness*) in the β-band at T0 were significantly different between the two groups. The connectivity and network measures at T0 also appear to have a predictive role in functional recovery (BI T1-T0), again group-dependent. The results obtained in this study showed that connectivity measures and correlations between EEG features and recovery depend on lesion location. These data, if confirmed in further studies, on the one hand could explain the heterogeneity of results so far observed in previous studies, on the other hand they could be used by researchers as biomarkers predicting spontaneous recovery, to select homogenous groups of patients for the inclusion in clinical trials.

## Introduction

Stroke is a leading cause of severe long-term disability in both the United States (Benjamin et al., 2018) and Europe (source)^1^.

Ischemic stroke damages the brain tissue in the affected vascular territories, inducing a corresponding loss of function. The structural lesion resulting from unilateral brain injury affects the functional network architecture of the whole brain, causing early modifications of its electrical activity (Grefkes and Fink, 2011; Assenza et al., 2013; Fanciullacci et al., 2017) and a subsequent modification of functional connectivity both locally and remotely with respect to the lesion (Rossini et al., 2003; Caliandro et al., 2017). Due to the complexity of the brain response to neurologic injury, the outcome of stroke recovery is not only related to the extent of the initial damage (Chen et al., 2000; Alexander et al., 2010), but also depends on the results of brain plasticity processes, both structural and functional (Lin et al., 2018; Pichiorri et al., 2018).

How stroke affects the electrical activity and the synchrony of electrical oscillations in the cerebral neural network is still largely unknown (Dubovik et al., 2012), and how these changes are associated with neurological deficits is unclear. Recent studies have shown that the activity of individual hemispheres plays a key role in recovery after a unilateral stroke. In particular, a relationship between interhemispheric activity balance and functional recovery was demonstrated (van Putten, 2007; Agius Anastasi et al., 2017) and a different involvement of the affected and unaffected hemispheres (AH and UH, respectively) in different post-stroke phases was also highlighted (Murase et al., 2004; Sheorajpanday et al., 2011). In this regard, recent studies have described a specific brain reorganization in the UH that tends to interact with functional recovery in patients in the early post stroke stage (Van Kaam et al., 2018). Furthermore, the UH appears to be involved in a continuous support function even long after the injury (Lotze et al., 2006; Riecker et al., 2010; Finnigan and van Putten, 2013).

In addition to the activity of individual hemispheres, the involvement of specific brain structures was also investigated to characterize the clinical behavior after stroke. In this regards, preclinical studies have demonstrated that lesion location influences the degree of spontaneous recovery in murine models of cortical and subcortical stroke (Karthikeyan et al., 2019). Furthermore, in a recent longitudinal study, cortical involvement was found to predict a poorer functional outcome in subacute stroke patients (Qu et al., 2018). Lesion location has also been correlated to the onset of specific neuropsychiatric conditions after stroke, such as apathy and depression, which can severely affect recovery (Mihalov et al., 2016).

Recent findings based on TMS, fMRI and EEG studies pointed out that cortico-subcortical and subcortical stroke patients have different cortical activity and excitability levels of the motor cortex (Luft et al., 2004; Thickbroom et al., 2015; Lamola et al., 2016; Fanciullacci et al., 2017; Sarasso et al., 2020). However, the impact of these differences on functional outcome has not been fully elucidated yet.

The brain is a complex, interconnected network. Pathological perturbations of the brain are rarely confined to a single locus; instead, they often spread via axonal pathways to influence other regions. Understanding brain disorders requires knowledge of how brain networks respond to pathological perturbations (Alstott et al., 2009). For this reason, in addition to standard quantitative EEG analyses, more advanced tools from graph theory (Grefkes and Fink, 2011) have also been used in recent years to describe brain function from a systemic perspective, an approach known as Connectomics (Silasi and Murphy, 2014). Graph theory looks at networks as a set of nodes with links between them. Connectomics describes the links in the network by means of brain connectivity measures.

For EEG recordings of brain network activity, the nodes are the electrodes/sensors or reconstructed regions of interest (ROIs), and the links can be considered the connections between them, calculated with techniques such as linear or nonlinear correlation, coherence, causality index (Astolfi et al., 2006; Marinazzo et al., 2011; Jovanović et al., 2013; Guggisberg et al., 2019). A graph type that shows both “good” local connections and some distant connections can be called a “small world network” (Watts and Strogatz, 1998). Small-world organization of brain networks has been investigated also after stroke (Wang et al., 2010; Caliandro et al., 2017), and this and other measures from graph theory have been used to quantify the effects of changes in brain connectivity on clinical impairment and functional recovery of post-stroke patients (Jiang et al., 2013; Rehme and Grefkes, 2013; Wu et al., 2015; Van Kaam et al., 2018).

The aim of this study is to use both standard and more advanced methods for characterizing resting state EEG activity and functional connectivity changes in a cohort of patients during the sub-acute phase of unilateral ischemic brain injury. Our focus is to understand the dependence of these modifications on lesion type (cortico-subcortical vs. subcortical). In detail, we hypothesize a specific effect of the type of lesion on cortical electrical activity and network connectivity, and a predictive role of the different early reorganization in functional recovery over time.

## Materials and Methods

### Participants

Thirty-three unilateral ischemic stroke patients (mean age 69 years, range 22–85; 21 males and 12 females) were enrolled at the Neurorehabilitation Unit of the University Hospital of Pisa in the subacute phase of the disease (between 10 and 45 days after the stroke). Inclusion criteria were: (1) age between 18 and 80 years; (2) first-ever unilateral ischemic stroke in the middle cerebral artery territory; (3) time from acute event within 45 days. Exclusion criteria were: (1) use of drugs targeting CNS; (2) diagnosis of epilepsy; and (3) Mini-Mental State Examination (MMSE) <24. Brain injury was assessed by means of a standard Computed Tomography (CT) scan, performed in the Neuroradiology Department of the University Hospital of Pisa. Based on brain CT images, lesions were defined as subcortical if they involved the deep white matter inferior to the corpus callosum, including the internal capsule, thalamus, basal ganglia and spared the cerebral cortex. All lesions with a cortical involvement were defined as cortical-subcortical. According to the lesion site, the initial sample was divided into two groups: 18 with cortical-subcortical (**CS**) and 15 with subcortical (**S**) lesions. Mean (range) age of **S** patients was 66 (22–82) years; mean (range) age of **CS** patients was 72 (54–85) years. The first clinical evaluation (T0) was performed after 23 days on average (range 11–45) for **S** patients, and after 26 days (range 14–45) for **CS** patients by trained neurologists. Patients were evaluated again 3 months after the acute event (T1). The Barthel Index (BI) was used to measure functional autonomy. Data about age, gender, side of the lesion (right or left hemisphere), stroke location, time since stroke at the time of evaluation and clinical status for all the patients are reported in Table 1. Ten neurologically intact age-matched right-handed subjects (M/F: 4/6; mean age ± SD: 62.0 ± 10.3 years) were also included in the study as control group. Each patient and healthy subject recruited gave written informed consent in accordance with the Declaration of Helsinki. This study was authorized by the local Ethics committee of Area Vasta Nord Ovest (CEAVNO) for Clinical experimentation, Tuscany (Italy), protocol n. 901.

**TABLE 1:** Participant characteristics.

**Patient**	**Age**	**Gender**	**Lesion side/Location**	**Lesion site**	**T0 (days)**	**BI (T0)**	**BI (T1)**
1	54	M	R/Temporoparietal	CS	31	5	30
2	65	M	R/Frontotemporal	CS	15	5	35
3	79	F	L/Temporal	CS	23	0	30
4	85	M	L/Frontal	CS	45	0	15
5	76	M	R/Frontal	CS	26	25	60
6	80	F	L/Parietal	CS	45	5	30
7	77	F	R/Parietal	CS	14	10	20
8	73	F	R/Frontotemporoparietal	CS	34	10	55
9	61	F	R/Parietal	CS	14	85	85
10	61	F	L/Frontal	CS	10	94	98
11	66	M	L/Temporoparietal	CS	19	5	45
12	69	F	L/Temporal	CS	17	100	100
13	67	M	R/Temporal	CS	23	90	95
14	67	M	R/Frontotemporal	CS	45	5	N/A
15	84	F	R/Temporal	CS	23	0	N/A
16	83	F	L/Parietotemporal	CS	28	0	N/A
17	59	M	R/Parietal	CS	19	90	N/A
18	85	F	R/Frontotemporal	CS	43	0	N/A
19	54	M	R/Corona radiata	S	22	35	65
20	59	M	L/Pons	S	45	10	20
21	78	M	L/Centrum semiovale	S	19	30	75
22	63	M	R/Corona radiata	S	19	30	55
23	65	M	L/Centrum semiovale	S	32	35	65
24	22	M	L/Insula	S	21	95	100
25	82	F	R/Lenticulostriate	S	27	20	30
26	52	M	L/Corona radiata	S	15	100	100
27	73	M	R/Centrum semiovale	S	11	55	75
28	80	M	R/Insula	S	18	20	50
29	77	M	R/Pons	S	20	50	85
30	71	M	L/Internal capsule	S	45	10	N/A
31	70	M	L/Internal capsule	S	23	15	N/A
32	62	M	R/Thalamus	S	11	15	N/A
33	82	F	R/Internal capsule	S	45	0	N/A

*Gender: M, male; F, female. Lesion side: R, right; L, left. Lesion site: CS, cortico-subcortical; S, subcortical. Timing at T0 is the number of days after the stroke event. BI, Barthel Index.*

### Electrophysiological Recordings and Data Preprocessing

EEG recordings lasting 10 min were performed at rest while the subjects were seated in a comfortable chair, with eyes closed, in an acoustically and electrically shielded room. The EEG and the vertical electrooculogram (EOG) were recorded using a 64-channel DC-coupled monopolar amplifier (Micromed SD MRI, System Plus acquisition software). Electrodes were positioned according to the 5% 10/20 system (Oostenveld and Praamstra, 2001). After careful scalp preparation, EEG signals were acquired at a sampling rate of 256 Hz. Skin/electrode impedances were below 10 kΩ in at least 95% of derivations throughout the experiment (electrode re-gelling was performed whenever required). Data containing artifacts due to eye blinks, significant muscle activity and electrode displacement were removed in an offline visual screening. Although the influence of ocular artifacts with eyes closed is lower than with eyes open, small ocular movements may still be present. Given their stereotyped nature, independent component analysis (ICA) enabled us to identify and remove such residual ocular activity if present. The EEG signals were offline re-referenced to the linked-mastoids virtual reference, then high-pass filtered with a zero-phase Chebyshev type-2 filter (1 Hz stopband, 2 Hz passband, 80 dB attenuation) and low-pass filtered with a Chebyshev type 2 filter (45 Hz passband, 48 Hz stopband, and 80 dB attenuation). Abnormal data with extreme magnitude (e.g., mean deviations, jumps and large oscillations) with respect to the continuous dataset were removed by using a customized version of the routine *flt_clean_windows* (BCIlab) to compute a moving windowed signal power. EEG windowed segments (1s) were removed if their power exceeded the 90% distribution quantile. Synchronous sudden increases in signal amplitude were detected by computing the difference between the superior and inferior envelopes (shape-preserving piecewise cubic interpolation; Butt and Brodlie, 1993), EEG portions with values greater than 2.5 standard deviations (in amplitude distribution) were then removed. The process followed three consecutive steps: (i) the computation of inferior and superior all-channels envelopes; (ii) the local range setting as all-channels envelopes, respectively; and (iii) the detection of movement artifacts related to a suitable threshold on the local range (2.5 standard deviations in amplitude distribution; Artoni et al., 2012b). Bad channels were identified by computing global channel measures (e.g., Kurtosis), and by visual inspection. Eye blinks were identified by computing an adaptive threshold on the moving-windows cross-correlation between the EOG and the frontal EEG channels (Menicucci et al., 2014; Sebastiani et al., 2015; Genna et al., 2017). Finally, ICA was applied on the cleaned dataset and the ocular component was removed (Artoni et al., 2012a, 2014).

### EEG Data Analysis

#### Power Analysis

Power spectral density (PSD) was computed for each channel by averaging periodograms of windowed signal sections (*pwelch* function in Matlab). The window length was 2s (512 time points), without zero padding or overlap. On average 8–9 min of artifact-free EEG data per patient were available for power analyses. The PSD was computed for the unaffected hemisphere (UH) and for the affected hemisphere (AH). An “average scalp power spectrum” was defined as the mean PSD across all scalp electrodes. From the average scalp power spectra, we computed the average PSD across the following frequency bands: delta (2–4 Hz), theta (4–8 Hz), alpha (8–14 Hz) and beta (14–30 Hz). PSD was also computed on 26 cortical regions of interest (ROIs) reconstructed from the EEG signals (see section “Brain Sources Reconstruction”), using the same methods as for the PSD computation at the electrode level. The power in the frequency bands of interest was estimated for each ROI by computing the area under the PSD curve.

#### Brain Sources Reconstruction

Underlying brain source signals were determined by processing IC-reconstructed scalp EEG data using source localization functions in the eConnectome Matlab toolbox (He et al., 2011). Relevant steps are explained in He et al. (2011) and in Artoni et al. (2017) and summarized here for convenience. A cortical current density source model was used to solve the inverse problem from the artifact-pruned and retained IC-reconstructed scalp EEG to cortical source distribution using the minimum norm estimate (MNE) with the aid of a boundary element method (BEM) forward head model (He et al., 1987; Hamalainen and Sarvas, 1989) and Tikhonov regularization (Hansen, 2007). A high-resolution cortical surface consisting of 41,136 triangles, segmented and reconstructed for visualization from MRI images of the Montreal Neurological Institute (MNI) brain using the Curry software (NeuroScan, Charlotte, NC, United States) was used. A source space was formed from this cortical surface down-sampled to 7,850 voxel dipole locations, constrained to the gray matter with orientations perpendicular to the containing cortical surface voxel. The scalp surface, skull surface and brain surface, segmented and reconstructed from the MNI brain, are provided by the toolbox. The scalp surface, which consists of 2,054 triangular voxels, forms the sensor space. This approach enables the comparison of source localizations across subjects in an atlas-based coordinate system that can be used in most EEG studies in which subject MR head images are not available (Darvas et al., 2006; Valdes-Hernandez et al., 2009). With the pre-computed high-resolution lead field matrix (2,054 × 7,850) relating all the scalp surface voxels to the source voxels, a specific lead field matrix for a user-defined electrode montage (standard 10–20 System in this case) can be constructed as a subset of the pre-computed lead field matrix and then used to solve an inverse problem. The solution of the inverse problem yields estimates of continuous time courses for cortical sources. Cortical regions of interest (ROI) can be defined according to Brodmann areas. Thirteen ROIs were defined for each cortical hemisphere: parietal cortex, associative area (BA5), ventral premotor cortex (BA6), dorsal premotor cortex (BA6a), occipital lobe, visuo-motor coordination (BA7), frontal cortex (BA8), prefrontal cortex (BA9_46), occipital cortex (BA19), supplementary motor (SMAp), cingulate motor cortex (CMA), primary motor cortex, BA4, divided into foot area (MIF), lip area (MIL), hand area (MIH), and the primary somatosensory cortex, BA3, hand representation area (SIH). Each ROI source signal was computed by averaging estimated cortical source activities across the source space ROI voxels. It is important to point out though that estimating the actual precision of source localization is currently an open research field (Akalin Acar et al., 2016). Even when inverse source solutions are estimated (either as cortical patches or their equivalent dipoles) using an electrical head model incorporating individual (or template) head tissue geometries and generally assumed conductivity values, the resulting inverse source localization should be interpreted “probabilistically” (Bigdely-Shamlo et al., 2013), with the spatial confidence boundaries (of, in general, a cm or more) difficult to estimate. In the case of distributed cortical surface estimates, the size of the estimated source patch, in particular, may be highly method-dependent.

#### Functional Connectivity Analysis

In this work, measures of lagged connectivity were preferred to zero phase-lag coherence-type metrics as they proved to be less biased and less affected by volume conduction effects (Pascual-Marqui et al., 2011).

Formally, let *X*_i_(*t*),*Y*_i_(*t*) ∈ *R*_3 ×1_ denote the three-dimensional time series for the current density vectors (i.e., the intracranial signals of electric neuronal activity) at any two voxels, for the i  -th recording epoch, at time t. The complex-valued Fourier transforms at frequency ω are denoted *X*_i_(ω),*Y*_i_(ω) ∈ *C*_3 ×1_.

For *N* recording epochs, the complex-valued covariance can be written in partitioned form as:

(1) $$S\,\left(\omega \right)=\left(\begin{array}{cc} S_{\text{YY}}\,\left(\omega \right) & S_{\text{YX}}\,\left(\omega \right)\\ S_{\text{XY}}\,\left(\omega \right) & S_{\text{XX}}\,\left(\omega \right) \end{array}\right)=\frac{1}{N}\,\sum_{i=1}^{N}Z_{\text{i}}\,\left(\omega \right)\cdot Z_{\text{i}}^{*}\,\left(\omega \right)$$

with:

(2) $$Z_{\text{i}}\,\left(\omega \right)=\left(\begin{array}{c} X_{\text{i}}\,\left(\omega \right)\\ Y_{\text{i}}\,\left(\omega \right) \end{array}\right)$$

and where the superscript “^∗^” denotes the transposed and complex conjugate vector. The partitioned coherence matrix which conserves each intra-voxel structure is:

(3) $$\text{R}\,\left(\omega \right)=\left(\begin{array}{cc} S_{\text{YY}}^{-1/2}\,\left(\omega \right) & 0\\ 0 & S_{\text{XX}}^{-1/2}\,\left(\omega \right) \end{array}\right)\,\left(\begin{array}{cc} S_{\text{YY}}\,\left(\omega \right) & S_{\text{YX}}\,\left(\omega \right)\\ S_{\text{XY}}\,\left(\omega \right) & S_{\text{XX}}\,\left(\omega \right) \end{array}\right)\,\left(\begin{array}{cc} S_{\text{YY}}^{-1/2}\,\left(\omega \right) & 0\\ 0 & S_{\text{XX}}^{-1/2}\,\left(\omega \right) \end{array}\right)$$

Finally, as explained in detail in Pascual-Marqui et al. (2011), the difference between the appropriate statistics for testing total and instantaneous connectivities gives the well-defined measure of lagged connectivity:

(4) $$F_{X↔Y}\,\left(\omega \right)=\ln\left|R\,e\,\left(\text{R}\,\left(\omega \right)\right)\right|-\ln\left|\text{R}\,\left(\omega \right)\right|$$

or its transformation as lagged coherence:

(5) $$r_{\text{lag}}^{2}\,\left(\omega \right)=1-\text{exp}\,\left(-{\text{F}}_{\text{lag}}\,\left(\omega \right)\right)$$

In the particular case for univariate time series *x*_i_(*t*) and *y*_i_(*t*), the lagged coherence has a very simple and appealing form:

(6) $$r_{\text{lag}}^{2}\,\left(\omega \right)=\frac{{\left[s_{\text{xy}}^{I\,m}\,\left(\omega \right)\right]}^{2}}{s_{\text{xx}}\,\left(\omega \right)\,s_{\text{yy}}\,\left(\omega \right)-{\left[s_{\text{xy}}^{R\,e}\,\left(\omega \right)\right]}^{2}}$$

where the superscripts *Re* and *Im* denote real and imaginary parts, respectively.

It is shown in Pascual-Marqui (2007) and Pascual-Marqui et al. (2011) that the lagged connectivity measure for intracranial signals contains physiological information, minimally affected by volume conduction artifacts. Furthermore, note that while coherence quantifies the linear relationship between complex-valued variables, lagged coherence measures exactly the same, but with the exclusion of zero-lag contribution.

In this study, in order to explore interconnectivity between the reconstructed ROIs, intracortical Lagged Linear Coherence was computed between all possible pairs of ROIs for each of the independent EEG frequency bands of interest: delta (2–4 Hz), theta (4–8 Hz), alpha (8–14 Hz), and beta (14–30 Hz). Then, the following connectivity measures were extracted: *Global connectivity*, i.e., the average connectivity between all pairs of ROIs; *AH connectivity*, i.e., the average connectivity between ROIs in the affected hemisphere; *UH connectivity*, i.e., the average connectivity between ROIs in the unaffected hemisphere; *IH connectivity*, i.e., the average connectivity between ROIs located in different hemispheres.

### Network Analysis

A network is a mathematical representation of a real-world complex system and is defined by a collection of nodes (vertices) and links (edges) between pairs of nodes. Nodes in large-scale brain networks represent brain regions, while links represent anatomical or functional connections, depending on the data set. Anatomical connections typically correspond to white matter fiber tracts between pairs of gray matter brain regions (cortical areas or subcortical relays). Functional connections correspond to temporal correlations in activity and may also occur between pairs of anatomically unconnected regions. Connections between pairs of nodes in a network can be differentiated by assigning them weights, which measure their strength or intensity. In these cases, a weighted network can be created.

In the following network analyses, LLC values computed between all possible pairs of ROIs (nodes) were used as weighted links to create undirected weighted graphs, for each frequency band and for each subject separately. “Undirected” means that the link between node *i* and node *j* (forward connection) has the same strength of that between node *j* and node *i* (backward connection).

We computed the core measures of graph theory that summarize the aspects of segregation and integration of a network. *Segregation* (or specialization) refers to the degree to which network elements form individual and separate clusters of nodes; the tendency of nodes to be organized in clusters is measured by the clustering coefficient (*C*). *Integration* refers to the capacity of the network as a whole to become interconnected and exchange information; the level of integration is measured by the characteristic path length (*L*).

Since in the present study weighted brain networks were analyzed, the weighted clustering coefficient (*C*_w_) and the weighted characteristic path length (*L*_w_) were computed as a measure of segregation and integration.

The weighted clustering coefficient of a single node is defined as:

(7) $$\hat{C_{\text{i}}}=\frac{2}{k_{\text{i}}\,\left(k_{\text{i}}-1\right)}\,\sum_{j,k}{\left({\hat{w}}_{\text{ij}}\,{\hat{w}}_{\text{jk}}\,{\hat{w}}_{\text{ki}}\right)}^{1/3}$$

where *k*_i_ is the degree of node *i*, and the weights are scaled by the largest weight in the network, i.e., w̨_ij_ = *w*_ij_/*max*⁡(*w*_ij_), where *max*⁡(*w*_ij_) represents the value of the edge with the largest weight in the network.

The mean weighted clustering coefficient (*C*_w_) is computed by averaging the weighted clustering coefficient of all single nodes ($\hat{C_{l}}$) in the network. *C*_w_ is a measure for the tendency of the network to be organized in local clusters.

For unweighted networks (Watts and Strogatz, 1998), the characteristic path length is defined as:

(8) $$L=\frac{1}{n}\,\sum_{i\in N}L_{\text{i}}=\frac{1}{n}\,\sum_{i\in N}\frac{\sum_{j\in N,j\neq i}d_{\text{ij}}}{n-1}$$

where *L*_i_ is the average distance between node *i* and all other nodes; *d*_ij_ the shortest path length between nodes i and j, defined as:

(9) $$d_{\text{ij}}=\sum_{a_{u\,v}\in g_{i↔j}}a_{u\,v}$$

where *g*_*i↔j*_ is the shortest path (geodesic) between *i* and *j*.

The weighted characteristic path length (*L*_w_) is thus defined as:

(10) $$L_{\text{w}}=\frac{1}{n}\,\sum_{i\in N}\frac{\sum_{j\in N,j\neq i}d_{\text{ij}}^{w}}{n-1}$$

where $d_{\text{ij}}^{w}=\sum_{a_{u\,v}\in g_{i\to j}}f\,\left(w_{u\,v}\right)$ represents the shortest weighted path length between i and *j*. f is a map (e.g., an inverse) from weight to length, and *g*_*i↔j*_ is the shortest weighted path between *i* and j. In other words, *L*_w_ is the average of the shortest and contemporary highest-weighted paths connecting each node to all the other ones. In each subject, we normalized the values of *C*_w_ and *L*_w_ of each band versus the respective mean values obtained averaging each parameter through all the bands.

Originally described in social networks, the “small-world” property combines high levels of local clustering among nodes of a network (to form families or cliques) and short paths that globally link all nodes of the network. This means that all nodes of a large system are linked through relatively few intermediate steps, even though most nodes maintain only a few direct connections—mostly within a clique of neighbors. The measure of network small-worldness, *S*, is defined as the ratio between the weighted clustering coefficient and the characteristic path length. For weighted networks, the weighted small-worldness, *S*_w_, can be thus defined as:

(11) $$S_{\text{w}}=\frac{C_{\text{w}}}{L_{\text{w}}}$$

where *C*_w_ and *L*_w_ are the weighted clustering coefficient and the weighted characteristic path length, respectively, individually normalized with respect to the frequency bands. The *S*_w_ coefficient is used to describe the balance between the local connectedness and the global integration of a network. Small-world organization mixes short path length (typical of random networks) and high clustering (typical of regular networks). When *S*_w_ is larger than 1, a network is said to have small-world properties.

### Statistical Evaluation

The Shapiro–Wilk test was used to assess whether variables were normally distributed. Group effect at single time points (T0 or T1) was evaluated by means of a Kruskal–Wallis test. *Post hoc* analyses (Mann-Whitney *U* tests) were performed to compare groups, with Bonferroni correction. The Wilcoxon signed-rank test was used to compare BI scores at T0 with scores at T1 in each patient group. Spearman correlation analyses were performed between clinical measures and relevant electrophysiological variables. Correction for multiple comparisons was applied to control the FDR as explained in Benjamini and Hochberg (1995). Statistical analysis was performed with SPSS 20.0 software (SPSS Inc., Chicago, IL, United States). Significance of statistical tests was set at α = 0.05.

## Results

### Clinical Evaluation

**FIGURE 1:**
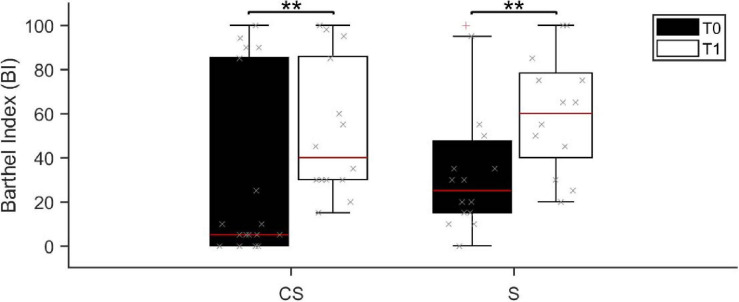
Barthel Index score assessed at baseline (T0, black boxes) and at the 3 months follow-up (T1, white boxes). BI, Barthel Index; **CS**, cortico-subcortical stroke patients; **S**, subcortical stroke patients. **p < 0.01.

Patients in the **CS** group presented a similar global functional status (assessed by BI) than patients in the **S** group, both at admission (median BI score was 5 range 0–100 in **CS** and 30 range 0–100 in **S**; *U* = 85.0, *p* = 0.073) and in the follow up (median BI score was 40 range 15–100 in **CS** and 65 range 20–100 in **S**; *U* = 73.0, *p* = 0.402). Age and Timing of patients’ first assessment was not significantly different between the two patient groups (Age: *U* = 107.0, *p* = 0.32; Timing: *U* = 125.5, *p* = 0.735). Barthel Index at T0 was negatively correlated with both age (*R* = −0.563, *p* = 0.001) and timing of patients’ first assessment after the acute event (*R* = −0.532, *p* = 0.002). Despite the functional status was largely variable at T0, 3 months after the acute event (T1) the BI score showed a statistically significant increase in both patient groups (**CS:** *Z* = −3.065, *p* = 0.002; **S:** *Z* = −3.070, *p* = 0.002; Figure 1), meaning that patients reached a higher degree of independence, on average. Information on individual patients is presented in Table 1. Twenty-four (thirteen **CS** and eleven **S**) of the initial thirty-three patients were available to be reassessed at T1.

### Power Spectral Density Analysis of EEG Signals

**FIGURE 2:**
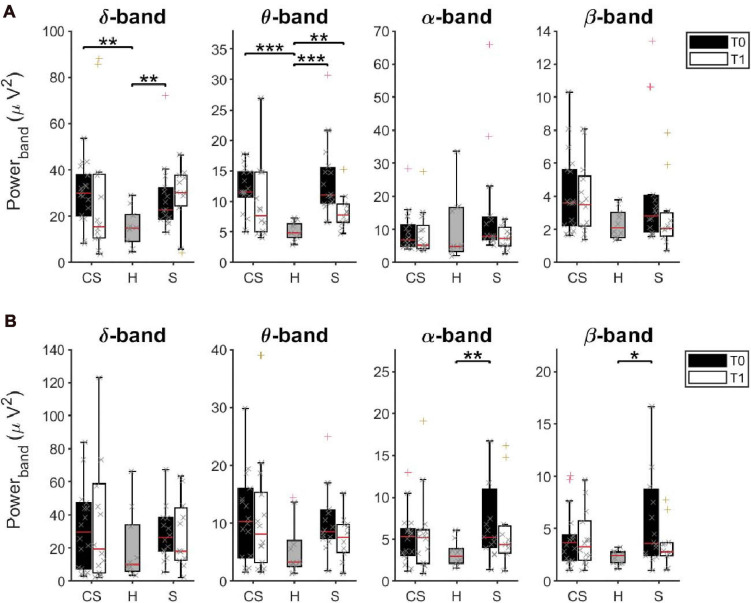
Average Power Spectral Density of EEG sensors **(A)** and of reconstructed ROIs **(B)** in δ, θ, α, and β bands at T0 and T1, respectively, for cortico-subcortical (*CS*) patients, subcortical (*S*) patients and healthy subjects (*H*). *p < 0.05, **p < 0.01, ***p < 0.001.

The power spectral density was calculated for signals from both the EEG sensors and the reconstructed sources (ROIs), separately, from EEG data recorded at rest with eyes closed (Figure 2).

#### EEG Sensors

The results of a multivariate Kruskal–Wallis test showed a main effect of factor *Group* on average PSD values of the δ-band [H(2) = 10.745, *p* = 0.005] and of the θ-band [H(2) = 19.619, *p* < 0.001] but not of the other frequency bands. Specifically, as revealed by *post hoc* analyses, both **CS** and **S** patients showed significantly increased power, compared to healthy subjects (**H**) in these two bands (δ-band, **CS** vs. **H:** *U* = 28.0, *p* = 0.002; **S** vs. **H:** *U* = 27.0, *p* = 0.007; θ-band, **CS** vs. **H:** *U* = 9.0, *p* < 0.001, **S** vs. **H:** *U* = 2.0, *p* < 0.001).

Similar results were found when comparing PSD values only in the affected hemisphere (AH) or in the unaffected hemisphere (UH) between groups.

When tested again after 3 months (T1), significant differences were still found between groups in the θ-band [H(2) = 8.947, *p* = 0.011]. Specifically, PSD values of the θ-band of **S** patients were still significantly higher than in healthy subjects (**S** vs. **H:** *U* = 14.0, *p* = 0.003). No statistical differences were found in the other frequency bands.

We investigated whether the initial (T0) and residual (T1) increase of θ-band power in stroke patients could be linked to a slowing of alpha frequency near theta ranges. We thus estimated the Individual Alpha Frequency (IAF) for each patient by averaging the center of gravity of each EEG channel in the alpha band (8–14 Hz) from the power spectral density (Klimesch et al., 1990). The Kruskal–Wallis test showed a main effect of factor *Group* on average IAF values at T0 [H(2) = 9.765, *p* = 0.008] but not at T1 [H(2) = 4.479, *p* = 0.106]. However, the slowdown of alpha frequency at T0 was significant only in **S** patients compared to healthy subjects (*U* = 26.0, *p* = 0.005), and recovered at T1, unlike the increase in power in the θ-band.

#### ROIs Activity

Interestingly, the multivariate Kruskal–Wallis test on PSD values from ROIs showed complementary results. A main effect of factor *Group* was found on average PSD values of the α-band [H(2) = 6.698, *p* = 0.035] and of the β-band [H(2) = 7.318, *p* = 0.026] but not of the other frequency bands. Specifically, **S** patients had significantly increased α-rhythms and β–rhythms, compared to healthy subjects (α-band, **S** vs. **H:** *U* = 28.0, *p* = 0.008; β-band, **S** vs. **H:** *U* = 31.0, *p* = 0.014). No significant difference of average power was found at T1 between groups, in all frequency bands (*p* > 0.05).

#### Correlation Between PSD Values at T0 and BI Scores

This analysis investigated whether BI scores at T0 (BI T0) or BI scores variation (BI T1-T0) showed association with power of EEG frequency bands of interest computed at T0 (Table 2) both from EEG sensors and reconstructed ROIs.

**TABLE 2:** Spearman’s correlations between average power of EEG frequency bands of interest and Barthel Index at T0 (BI T0) or Barthel Index variation (BI T1-T0).

***CS***	***Sensors***				***ROIs***			
	**δ-*PSD***	**θ-*PSD***	**α-*PSD***	**β-*PSD***	**δ-*PSD***	**θ-*PSD***	**α-*PSD***	**β-*PSD***
***BI T0***	−0.259	−0.047	0.612*	0.383	−0.409	−0.444	−0.211	−0.254
***BI T1-T0***	−0.119	−0.341	−0.522	−0.148	0.033	−0.060	−0.102	−0.044
***S***								
***BI T0***	−0.175	0.014	−0.117	−0.188	0.335	0.285	0.169	0.081
***BI T1-T0***	−0.381	−0.256	0.250	0.306	−0.367	−0.351	−0.028	0.078

*p < 0.05 after correction for multiple comparisons. The bold values represent statistical significance.

In the **CS** group, the average power of the α-band computed from EEG sensors showed a significant positive correlation with BI T0 (*R* = 0.612, *p* = 0.007). Therefore, for patients with cortical involvement in the stroke lesion, a higher PSD value in the α-band corresponded to a higher degree of independence at admission. For patients with subcortical lesions, the α-band did not show any significant correlation with BI scores, as well as the other EEG frequency bands.

No significant correlations were found between BI scores and power of frequency bands of interest computed from the ROIs.

#### Topographic Representation of Resting State Activity

**FIGURE 3:**
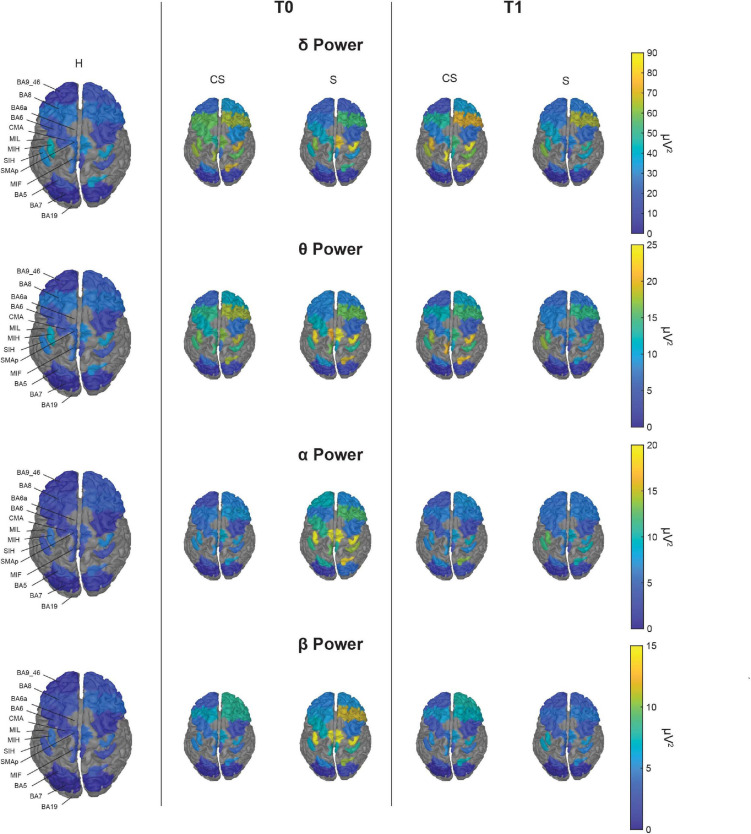
Topographic map of average power for the reconstructed ROIs at T0 (middle panels) and T1 (right panels) in each band of interest for the two group of patients (CS, cortico-subcortical and S, subcortical), compared to healthy subjects (H; left panels). For visualization purposes, ROIs BA19, BA6a, and MIL are not displayed as their mean power exceeds 3 median absolute deviations from the median value of all ROIs. The Kruskal–Wallis test was used to look for differences between the healthy group (H) and the stroke subgroups (CS and S) in each ROI. The significant differences found are shown in Figure 4.

**FIGURE 4:**
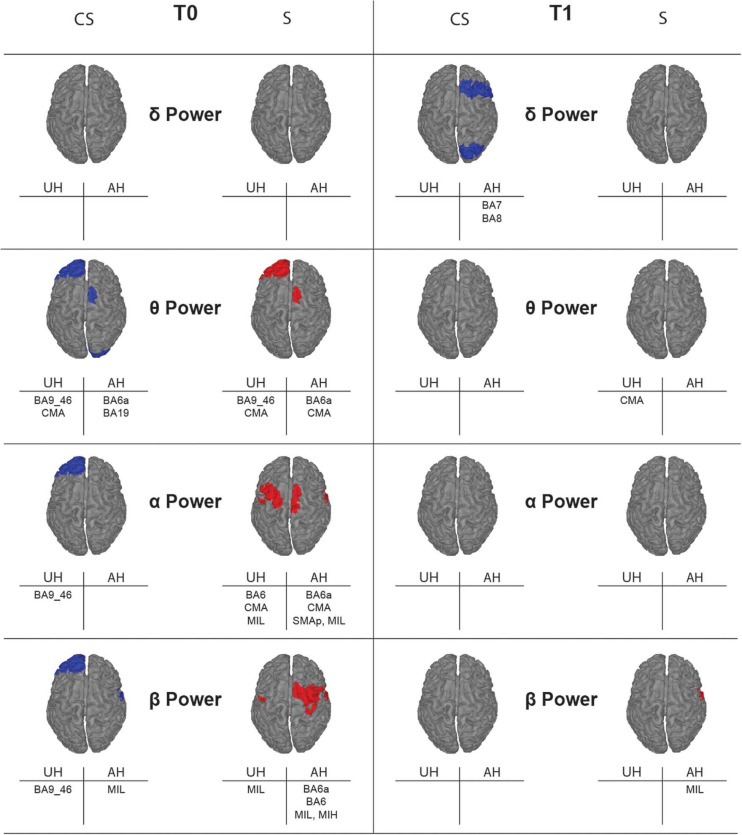
Topographic map showing significant differences of average power for the reconstructed ROIs between healthy subjects (*H*) and stroke patients (*CS*, cortico-subcortical and *S*, subcortical) at T0 (left panels) and T1 (right panels) in each band of interest. ROIs with significantly higher power (*p* < 0.05) in one of the stroke subgroups than in the healthy group are colored, in blue for *CS* and in red for *S*. Labels of significantly different ROIs are reported at the bottom of the corresponding image (for the correspondence between the ROI label and its full name see section “Materials and Methods”).

With illustrative purposes, we show in Figure 3 a topographic map of average PSD values for the reconstructed ROIs in the three groups (**CS**, **S**, and **H**), in each band of interest separately. Depending on the band of interest, specific ROIs display significantly different activity (i.e., average PSD) in healthy subjects (**H**) compared to stroke patients, both at T0 and T1 (Figure 4). Remarkably, in **S** patients, the power of dorsal premotor cortex (BA6a) in the affected hemisphere at T0 was significantly higher than in healthy subjects (for all bands except δ-band). On the other hand, the activity of the prefrontal cortex (BA9_46) in the unaffected hemisphere increased mainly in **CS** patients, compared to healthy subjects. The activity of both affected and unaffected hemispheres after 3 months almost completely normalized in both stroke patients.

#### Average Lagged Linear Connectivity

**FIGURE 5:**
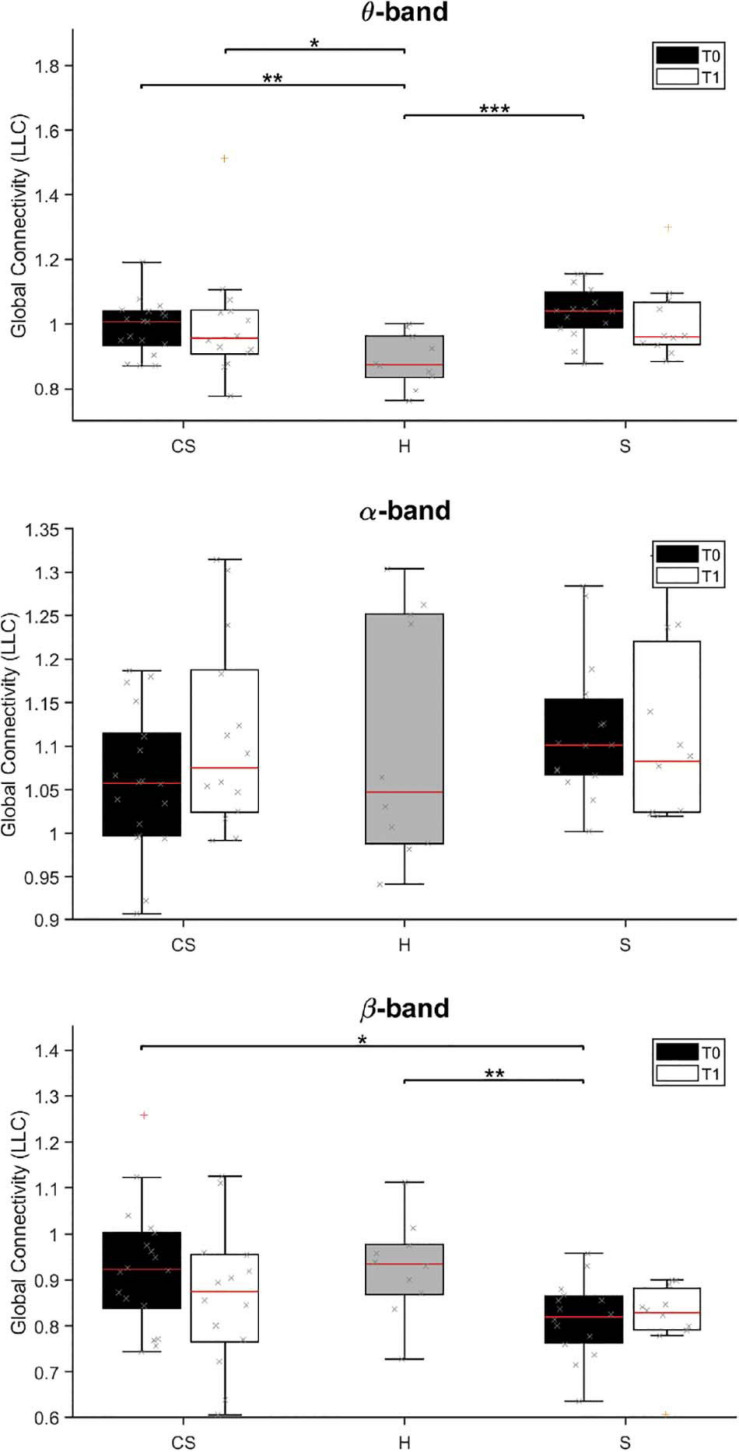
Lagged Linear Connectivity (LLC) values in θ-band (top panel), α-band (middle panel) and β-band (bottom panel) at T0 and T1 for cortico-subcortical (CS) and subcortical (S) patients, compared with LLC values in the group of healthy subjects (H). *p < 0.05, **p < 0.01, ***p < 0.001.

The results of brain connectivity analysis, using reconstructed ROIs, are shown in Figure 5. We used Lagged Linear Connectivity values to calculate a Global Connectivity index, two (partial) connectivity indexes for the affected (AH Connectivity) and the unaffected (UH Connectivity) hemispheres, and an interhemispheric connectivity index (IH Connectivity).

We found that average connectivity between ROIs across the whole brain (Global Connectivity) at T0 was significantly different between groups in the θ-band [H(2) = 14.205, *p* = 0.001] and β-band [H(2) = 8.702, *p* = 0.013]. Specifically, connectivity in the θ-band was lower in the **H** group than in both patient groups (**CS** vs. **H:** *U* = 32.0, *p* = 0.004; **S** vs. **H:** *U* = 13.0, *p* < 0.001), while connectivity in the β-band was lower in subcortical patients than in the other groups (**S** vs. **H:** *U* = 28.5, *p* = 0.008; **S** vs. **CS:** U = 66.0, *p* = 0.012). No between-group differences were found in the δ- and α-band (*p* > 0.05; δ-band data not shown).

At T1, Global Connectivity was still different between groups in the θ-band [H(2) = 6.602, *p* = 0.037]. Specifically, θ-band connectivity was still significantly higher in **CS** patients, compared to healthy subjects (**CS** vs. **H:** *U* = 21.0, *p* = 0.016).

Analysis of connectivity at T0 in the unaffected hemisphere (UH Connectivity) yielded non-significant differences across groups (*p* > 0.05, data not shown), whereas connectivity in the affected hemisphere (AH Connectivity) yielded similar results to Global connectivity for θ-band [H(2) = 16.831, *p* < 0.001; **CS** vs. **H:** *U* = 25.0, *p* = 0.001; **S** vs. **H:** *U* = 9.0, *p* < 0.001], but not for β-band [H(2) = 5.595, *p* = 0.061].

At T1, θ-band connectivity in the affected hemisphere still showed significant differences across groups [H(2) = 9.523, *p* = 0.009]. Specifically, connectivity was still significantly higher in both patient groups, compared to healthy subjects (**CS** vs. **H:** *U* = 29.0, *p* = 0.016; **S** vs. **H:** *U* = 14.0, *p* = 0.003).

Analysis of interhemispheric connectivity at T0 (IH Connectivity) showed a significant difference between groups in the θ-band [H(2) = 15.115, *p* = 0.001] and β-band [H(2) = 6.168, *p* = 0.046]. Specifically, interhemispheric connectivity in the θ-band was higher in both patient groups, compared to healthy subjects (**CS** vs. **H:** *U* = 29.5, *p* = 0.003; **S** vs. **H:** *U* = 10.0, *p* < 0.001), while interhemispheric connectivity in the β-band was lower in **S** patients than in **CS** patients (**S** vs. **CS:** *U* = 65.5, *p* = 0.011).

At T1, interhemispheric connectivity in both the θ-band and the β-band was no longer significantly different between patients and healthy subjects (*p* > 0.05).

#### Correlation of Average Connectivity at T0 With BI Scores

This analysis investigated whether BI scores at T0 (BI T0) or BI scores variation (BI T1-T0) showed association with the computed average connectivity measures at T0 (Table 3): *Global connectivity*, i.e., the average connectivity between all pairs of ROIs; *AH connectivity*, i.e., the average connectivity between ROIs in the affected hemisphere; *UH connectivity*, i.e., the average connectivity between ROIs in the unaffected hemisphere; *IH connectivity*, i.e., the average connectivity between ROIs located in different hemispheres.

**TABLE 3:** Spearman’s correlations between connectivity or network measures and Barthel Index (BI) at T0 or Barthel Index variation (BI T1-T0).

		***Connectivity***				***Network***		
***CS***	***ϑ***	***Glob T0***	***UH T0***	***AH T0***	***IH T0***	***INT T0***	***SEG T0***	***SW T0***
	***BI T0***	*0.298*	*0.245*	*0.283*	*0.267*	*−0.407*	*0.326*	*0.342*
	***BI T1-T0***	*−0.295*	*−0.181*	*−0.261*	*−0.246*	*0.268*	*−0.232*	*−0.221*
	**α**	***Glob T0***	***UH T0***	***AH T0***	***IH T0***	***INT T0***	***SEG T0***	***SW T0***
	***BI T0***	0.569*	0.567*	0.475*	0.539*	−0.479*	0.553*	0.514*
	***BI T1-T0***	−0.602*	−0.679*	*−*0.423	−0.584*	0.584*	−0.622*	−0.566*
	***β***	***Glob T0***	***UH T0***	***AH T0***	***IH T0***	***INT T0***	***SEG T0***	***SW T0***
	***BI T0***	−0.654*	−0.589*	−0.642*	−0.625*	0.629*	−0.658*	−0.654*
	***BI T1-T0***	0.533	0.528	0.471	0.505	*−*0.509	0.522	0.533

***S***	***ϑ***	***Glob T0***	***UH T0***	***AH T0***	***IH T0***	***INT T0***	***SEG T0***	***SW T0***

	***BI T0***	*−0.047*	*0.127*	*−0.094*	*−0.160*	*0.007*	*−0.098*	*−0.104*
	***BI T1-T0***	*0.167*	*0.175*	*0.118*	*0.070*	*−0.278*	*0.134*	*0.167*
	**α**	***Glob T0***	***UH T0***	***AH T0***	***IH T0***	***INT T0***	***SEG T0***	***SW T0***
	***BI T0***	*−*0.323	*−*0.117	*−*0.307	*−*0.326	0.380	*−*0.357	*−*0.364
	***BI T1-T0***	*−*0.267	−0.676*	*−*0.156	*−*0.264	0.289	*−*0.299	*−*0.298
	***β***	***Glob T0***	***UH T0***	***AH T0***	***IH T0***	***INT T0***	***SEG T0***	***SW T0***
	***BI T0***	*−*0.079	*−*0.382	0.098	*−*0.052	0.145	*−*0.069	*−*0.079
	***BI T1-T0***	0.245	0.504	0.100	0.200	−0.327	0.203	0.245

*p < 0.05 after correction for multiple comparisons. The bold values represent statistical significance.

We found a significantly positive correlation between BI T0 and all connectivity indices computed at T0 in the α-band of **CS** patients (Global Connectivity, *R* = 0.569, *p* = 0.014; AH Connectivity, *R* = 0.475, *p* = 0.046; UH Connectivity, *R* = 0.567, *p* = 0.014; IH Connectivity, *R* = 0.539, *p* = 0.021). Global Connectivity, UH Connectivity and IH Connectivity also showed a significant negative correlation with BI T1-T0 (*R* = −0.602, *p* = 0.023 and *R* = −0.679, *p* = 0.008, *R* = −0.584, *p* = 0.028, respectively). Therefore, for patients with cortical involvement in the stroke lesion, higher connectivity values in the α-band corresponded to higher degrees of independence at admission, but also to lower gains of independence over time (Table 3).

On the other hand, a significant negative correlation was found between BI T0 and all connectivity measures in the β-band (Global Connectivity: *R* = −0.654, *p* = 0.003; AH Connectivity: *R* = −0.642, *p* = 0.004; UH Connectivity: *R* = −0.589, *p* = 0.01; IH Connectivity: *R* = −0.625, *p* = 0.006). Therefore, contrary to the α-band, higher β-band connectivity values in **CS** patients corresponded to lower degrees of independence at admission (Table 3).

For **S** patients, on the other hand, non-significant correlations were found between most of the connectivity indices computed at T0 and the BI values (*p* > 0.05). Only a significant negative correlation was found between BI T1-T0 and connectivity of the unaffected hemisphere in the α-band (UH Connectivity, *R* = −0.676, *p* = 0.011). Therefore, for patients without cortical involvement in the stroke lesion, a higher β-band connectivity in the unaffected hemisphere was associated with a lower gain of independence in the activities of daily living over time.

### Network Connectivity Measures

#### Graph Analysis

**FIGURE 6:**
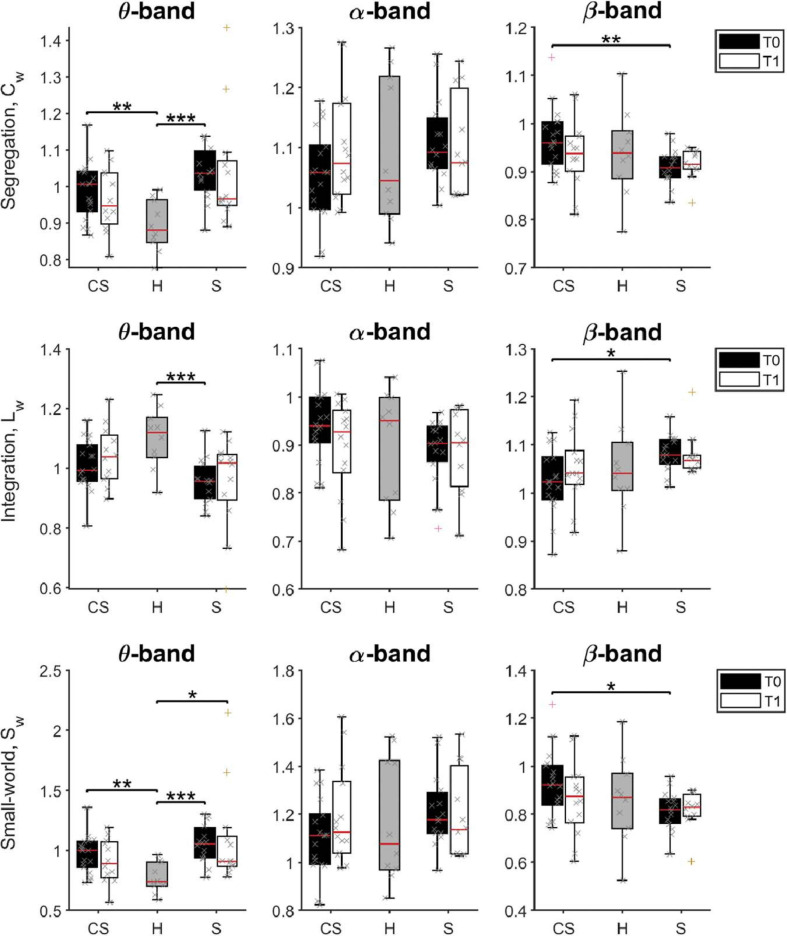
Pairwise comparison of network measures in θ-band (left panels), α-band (middle panels), and β-band (right panels) for cortico-subcortical (CS) patients, subcortical (S) patients and healthy subjects (H). Computed metrics are Weighted Clustering Coefficient (C_w_) for network segregation, Weighted Characteristic Path Length (L_w_) for network integration, and Weighted Small-Worldness (S_w_) for small-world network characteristics. *p < 0.05, **p < 0.01, ***p < 0.001.

Brain networks of **CS** and **S** patients and their changes overtime were also investigated with tools from graph theory. The mean weighted clustering coefficient, *C*_w_, the mean weighted characteristic path length, *L*_w_, and their ratio, *S*_w_ = *C*_w_/*L*_w_, were computed from connectivity values between all pairs of ROIs to measure brain network *segregation*, *integration*, and *small-worldness*, respectively, for all the frequency bands of interest (Figure 6).

We found that brain network *integration* (measured by the *L*_w_ parameter) at T0 was significantly different between groups in the θ-band [H(2) = 11.543, *p* = 0.003] and in the β-band [H(2) = 6.383, *p* = 0.041]. Specifically, network integration in the θ-band was lower in both patient groups, but was significantly lower compared to healthy subjects only for subcortical patients (**S** vs. **H:** *U* = 19.0, *p* = 0.001). Instead, network integration in the β-band was significantly different between the two patient groups, significantly higher in **S** patients than in **CS** patients (**S** vs. **CS:** *U* = 66.0, *p* = 0.012).

On the other hand, network *segregation* (measured by the *C*_w_ parameter) at T0 was also significantly different between groups in the θ-band [H(2) = 14.771, *p* = 0.001] and in the β-band [H(2) = 6.831, *p* = 0.033], but showed a different behavior. Specifically, network segregation in the θ-band was significantly higher in both patient groups, compared to healthy subjects (C**S** vs. **H:** *U* = 31.0, *p* = 0.004; **S** vs. **H:** *U* = 11.0, *p* < 0.001), whereas network segregation in the β-band was significantly lower in **S** patients than in **CS** patients (**S** vs. **CS:** U = 61.0, *p* = 0.007).

Finally, brain network *small-worldness* (*S*_w_) at T0 was also significantly different between groups in the θ-band [H(2) = 15.175, *p* = 0.001] and in the β-band [H(2) = 6.097, *p* = 0.047]. Specifically, network small-worldness in the θ-band was significantly higher in both patient groups, compared to healthy subjects (C**S** vs. **H:** *U* = 28.0, *p* = 0.002; **S** vs. **H:** *U* = 10.0, *p* < 0.001), whereas network small-worldness in the β-band was significantly lower in **S** patients than in **CS** patients (**S** vs. **CS:** *U* = 66.0, *p* = 0.012).

Therefore, the brain network of stroke patients at admission had, in the θ-band, lower connectivity between distant nodes (i.e., lower *L*_w_), each connected with a higher number of closer nodes (i.e., organized in a higher number of local clusters, a tendency measured by *C*_w_). This resulted in a significantly higher value for the small-world property of the θ-band network (small-world network: all node pairs can be linked in a few steps, despite most nodes maintain only direct connections with neighboring nodes).

β-band network measures showed instead a different brain network structure in the two patient groups soon after lesion: *C*_w_ was increased, while *L*_w_ decreased in the **CS** group compared to the **S** group. Consequently, the β-band network structure in **CS** patients had a higher *small-world* score compared to **S** patients at T0. However, no significant differences between patient groups and healthy subjects were found (*p* > 0.05).

At T1, *S*_w_ was still significantly different between groups in the θ-band [H(2) = 7.023, *p* = 0.030]. Specifically, network small-worldness in the θ-band was significantly higher in **S** patients compared to healthy subjects (**S** vs. **H:** *U* = 20.0, *p* = 0.013).

No significant differences of the brain network structure in the β-band were found between groups (*p* > 0.05).

#### Correlation of Network Measures With BI Scores

Finally, we investigated whether BI scores at T0 (BI T0) or BI scores variation (BI T1-T0) showed association with the computed brain network measures at T0 (Table 3).

In **S** patients, no significant correlations were found between BI scores and the computed brain network measures (Table 3).

In **CS** patients, instead, *C*_w_ and *S*_w_ computed in the α-band showed a significant positive correlation with BI T0 (*R* = 0.553, *p* = 0.017; *R* = 0.514, *p* = 0.029, respectively), while *L*_w_ showed an opposite behavior (*R* = −0.479, *p* = 0.044). Therefore, for patients with cortical involvement in the stroke lesion, a higher degree of independence at admission corresponded to higher *C*_w_ and lower *L*_w_ values, i.e., to a network with a higher small-world score (*S*_w_). In the β-band network, the association between BI T0 and the computed brain network measures at T0 was reversed: BI T0 was positively correlated with *L*_w_ (*R* = 0.629, *p* = 0.005), and negatively correlated with *C*_w_ (*R* = −0.658, *p* = 0.003). As a consequence, a higher degree of independence at admission corresponded to a network with a lower small-world score, *S*_w_ (R = −0.654, *p* = 0.003, respectively). No significant correlations were found with BI T0 by network measures in the other frequency bands (*p* > 0.05).

Network measures in the α-band at T0 were also correlated with BI variation over time. In fact, BI T1-T0 was positively correlated with *L*_w_ (*R* = 0.584, *p* = 0.028), and negatively correlated with *C*_w_ (*R* = −0.622, *p* = 0.018). Consequently, BI T1-T0 was also negatively correlated with *S*_w_ (*R* = −0.566, *p* = 0.035). Therefore, for patients with cortical involvement in the stroke lesion, a brain network at admission with a higher small-world score (*S*_w_) in the α-band was associated with a lower gain of independence in the activities of daily living over time.

## Discussion

The results obtained in this study demonstrated that brain electrical activity and connectivity are significantly modified by a stroke event, and such modifications depend on lesion location.

### Low-Frequency Activity

In our study, overall brain power computed from EEG sensors in the δ- and θ-bands was higher in stroke patients at T0 than in healthy controls, regardless of lesion site, consistently with previous literature (Murri et al., 1998; Tecchio et al., 2005, 2006; Assenza et al., 2013; Fanciullacci et al., 2017; Zappasodi et al., 2019). When considering the differences after 3 months, i.e., at T1, significant changes of whole brain electrical activity were detected only in the θ-band, and in stroke patients without cortical involvement.

Previous studies in humans have also shown that δ-band activity increases in correspondence of neural tissue damage, necrosis or both structural or functional deafferentation, and correlates with stroke lesion volume in the acute phase (Wu et al., 2016) as well as with a worse clinical status both in the acute and in the subacute phases (Assenza et al., 2009; Wu et al., 2016; Zappasodi et al., 2019). Conversely, an attenuation in θ power has been associated with greater disability in stroke survivors (Rogers et al., 2020). In our study, however, we could not reproduce these findings. Instead, analysis of brain power computed from ROIs revealed that baseline clinical status (BI T0) and low-frequency oscillations in stroke patients show an opposite (albeit non-significant) correlation depending on lesion location. Specifically, low frequency oscillations were negatively (positively) correlated with baseline clinical status in patients with (without) cortical involvement. These findings, if confirmed by further studies with greater samples, highlight the need for a stratification of stroke patients based on lesion location.

### α- and β-Band Activity

High oscillatory α-band activity has been associated with low brain metabolism (Moosmann et al., 2003). In line with this finding, we found a slight increase of α-band power in subcortical post-stroke subjects compared to healthy individuals in the early subacute phase, with a normalization in the late sub-acute phase. Thibaut et al. (2017) hypothesized that an increase in high-frequency EEG power could represent an attempt of cortical reorganization, leading to maladaptive plasticity. Similarly, we found that α-band power computed on ROIs was positively correlated with BI at T0 in **CS** patients, while there was a non-significant negative correlation with BI T1-T0. Moreover, in the same study, a decrease in corticospinal excitability, indexed by higher Resting Motor Threshold (RMT) values, was associated with a worse clinical status as well. Further studies should try to correlate different Transcranial Magnetic Stimulation (TMS) and EEG parameters to build combined markers to understand and predict stroke recovery.

When considering the relationship between α-power alteration and clinical status, Zappasodi et al. (2019) did not find any significant correlation between NIHSS and α-band power, both measured either in the acute phase (5 days) and in the sub-acute phase (2.5 months). However, Finnigan et al. (2007, 2016) and Leon-Carrion et al. (2009) showed that α-band rhythms could have a prognostic value, as relative α-band power measured after 48 h from the event was negatively correlated with the NIHSS score at 30 days, i.e., preservation (or even an increase) of α-activity in the acute phase corresponded to a higher outcome level after treatment. These discrepancies could be related to the variability of the signal recording, inherent of the high frequency bands, or to clinical variability such as timing of patients’ assessment after the acute event and different injury characteristics.

β-waves (14–30 Hz) seem to be related to the maintenance of the current sensorimotor or cognitive state, varying during cognitive information processing or physical activity (Engel and Fries, 2010; Rossiter et al., 2014). In our study, we found a difference in the β-band power, at T0, only between subcortical stroke patients and healthy subjects. Nevertheless, the relationship between abnormalities in β-power and impairment following stroke is still unclear. Despite the β-power decrease and the functional impairment are both driven by neuronal loss following ischemic stroke (Wu et al., 2016), previous studies showed a not direct correlation between β-activity and functional impairment, therefore it is considered not significant for ischemia monitoring (Finnigan et al., 2007). Our results confirm that β-power doesn’t correlate with clinical status. Instead, connectivity measures in the β-band could represent novel biomarkers of post-stroke recovery (see below).

### Functional Connectivity and Network Measures

Changes of the resting state network, as measured by the connectivity of reconstructed brain ROIs, were investigated with both measures of average brain functional connectivity and of network integration, segregation and small-worldness.

One of the main results obtained in our study are the differences in θ and β-band connectivity. Specifically, θ-band global connectivity was higher in both stroke subgroups than in healthy subjects, while β-band global connectivity was lower in the S group than in CS patients and healthy subjects. Previous literature showed an interaction trend between changes of remote functional connectivity and functional recovery (Wu et al., 2015; Van Kaam et al., 2018), but no direct involvement of the lesion site has been previously demonstrated.

Moreover, significant differences between brain network measures were detected in the θ-band and β-band. Specifically, network segregation was significantly higher, and network integration lower, in both groups of patients, compared to healthy subjects, leading to an increased small-worldness of the resting state network in the θ-band at T0. At T1, small-worldness in the θ-band was still higher in **S** patients compared to healthy subjects. Accordingly, Caliandro et al. (2017) found an increased segregation and a decreased integration in θ-band network, and these results are consistent with previous fMRI studies (Adhikari et al., 2017). On the other hand, β-band network measures were significantly different between stroke patients, with higher segregation and small-worldness in patients with cortical involvement. Moreover, in the same group of patients, a higher small-worldness in the α-band was predictive of poor recovery of autonomy in the activities of daily living.

Previous works have shown that brain networks of patients in both acute and subacute phases of stroke present rearrangements with respect to controls, which could be detected by measuring the network small-worldness (Wang et al., 2010; Tsirka et al., 2011; De Vico Fallani et al., 2012; Li et al., 2013; Caliandro et al., 2017). Small-worldness reflects an optimal network structure associated with rapid synchronization and information transfer, minimal wiring costs, as well as a balance between local processing and global integration (Watts and Strogatz, 1998). However, it is still not clear whether clinical recovery after stroke is paralleled by a decreased small-world organization of the brain (or parts of it), as suggested by some studies (Wang et al., 2010; Caliandro et al., 2017) or, on the contrary, by an increased small-worldness, as suggested by others (Tsirka et al., 2011; De Vico Fallani et al., 2012; Li et al., 2013). Several factors may in fact influence the evolution of the small-world parameter, such as patient cohort, phase of stroke, lesion type, brain areas investigated, brain signals (e.g., fMRI or EEG), band selected, etc. Further studies are thus required to investigate more in detail brain network rearrangements after stroke and their evolution with time and rehabilitation training.

Finally, we found that connectivity and network measures could represent novel biomarkers of post-stroke recovery. In fact, we found a positive correlation between α-band connectivity at T0 in both hemispheres and degree of independence in **CS** patients. However, when considering the BI changes over time, we found a clear negative correlation between baseline α-band connectivity in the UH and recovery. α-band dominates the activity of the awake brain and is a measure of the integrity of cortico-cortical and/or thalamo-cortical networks (Cantero et al., 2002). Therefore, changes in α-band connectivity are related to modifications of cortico-cortical and thalamo-cortical interactions which may have consequences on recovery after stroke. Contrary to what was found in α-band, β-band connectivity in both hemispheres and inter-hemispheric connectivity were inversely correlated with the degree of independence at T0. Conversely, a previous study by Pellegrino et al. (2012) has demonstrated that an increase in inter-hemispheric functional connectivity in the β-band between primary sensorimotor hand areas was associated with motor recovery following upper limb robotic rehabilitation treatment in chronic stroke patients. This discrepancy could be related to the different timing of patients’ recruitment. However, given its role in active motor control, investigating causal relationships between β-band activity and impairment represents an important progression in determining the clinical utility of EEG and may direct further interventional studies to facilitate greater functional recovery following stroke.

## Study Limitations

This study has some potential limitations. First, the sample was characterized by a variability in demographic and clinical aspects, such as age, stroke-event entity, brain areas involved by the ischemic injury. Results need thus to be confirmed in further studies with a greater sample to increase statistical power.

Another limitation regards the fact that power of brain rhythms was computed both at the electrode level and at the ROI level, whereas functional connectivity (FC) only at the ROI level. We initially discarded the idea of investigating FC in the sensor space, because it is well-known that the activity of an underlying EEG source typically spreads over more than one sensor due to volume conduction effects, thus limiting the usefulness of connectivity measures computed directly between sensor recordings (Schoffelen and Gross, 2009; Brunner et al., 2016; Lai et al., 2018; Van de Steen et al., 2019). Although recent approaches (Sanchez et al., 2018) have shown that sensor space FC analysis can still provide useful information if coupled with FC measures that minimize volume conduction effects, we preferred to focus on analyzing FC in the reconstructed source space. We would like to compare in greater depth sensor space and source space analyses in future works.

An additional limitation of our study is the use of an average brain model for source localization from EEG signals. On one hand, the absence of individual MRIs prevented us from obtaining a subject-specific source-space representation, thus reducing the accuracy of the analysis presented. On the other hand, this method ensures the consistency and homogeneity of ROIs reconstruction across subjects, and has already been widely used in the literature (Vecchio et al., 2019a,b).

Finally, the use of pre-defined ROIs (offered by the eConnectome software) may have limited the results of our study regarding potential contributions of temporal and occipital areas to the connectivity and network behavior of the brain after stroke. Moreover, the fine parcelization of the sensorimotor area may have given inaccurate results, due to the volume conduction effects and the lack of individual MR images.

## Conclusion

Our results suggest that brain modifications of electrical activity and connectivity are still present in the subacute stage of a stroke event and, together with their evolution with time, they depend on the lesion site in a frequency-dependent manner. Although brain power in the early subacute stage was similar in patients with cortico-subcortical or subcortical lesions, brain power remained significantly altered at T1 only in the subcortical group, and specifically in the θ-band. Moreover, the average connectivity and network measures were initially different between stroke patients and healthy subject (in the θ-band) or between the two stroke groups (in the β-band). Finally, the connectivity metrics also suggest their predictive role in functional recovery, still dependent on lesion location. Specifically, initial α-band average connectivity in the unaffected hemisphere negatively correlated with independence gain after 3 months in both patient groups. Whole-brain connectivity and network measures of segregation and small-worldness in the α- and β bands, however, also showed a predictive role, but only in patients with cortical involvement. Considered together, these results showed a clearcut role of lesion location on EEG features and provide complementary insights on the field of recovery biomarkers after stroke.

## Data Availability Statement

The raw data supporting the conclusions of this article will be made available by the authors, without undue reservation.

## Ethics Statement

The studies involving human participants were reviewed and approved by local Ethics Committee of Area Vasta Nord Ovest (CEAVNO). The patients/participants provided their written informed consent to participate in this study.

## Author Contributions

CF and AP recruited patients, performed neurophysiological evaluations, executed data analysis, interpreted the results, and drafted the manuscript. VS helped to interpret the results and draft the manuscript. ML and AM helped to perform data analysis and revised the manuscript. FA performed EEG signal preprocessing and revised the manuscript. SM was responsible for data analysis, revised the manuscript, and supervised the experiment. CC designed the study, was responsible for patients’ enrollment, supervised the experiment, contributed to interpretation of data, and revised the manuscript. All authors read and approved the final manuscript.

## Conflict of Interest

The authors declare that the research was conducted in the absence of any commercial or financial relationships that could be construed as a potential conflict of interest.

## Footnotes

1 www.escardio.org
